# Supplementary material for: Digital auscultation as a novel childhood pneumonia diagnostic tool for community clinics in Sylhet, Bangladesh: protocol for a cross-sectional study
Source: BMJ Open. 2022 Feb 9;12(2):e059630. doi: 10.1136/bmjopen-2021-059630 (PMC8830242; doi:10.1136/bmjopen-2021-059630)
Supplement: Reviewer comments [file bmjopen-2021-059630.reviewer_comments.pdf]

PEER REVIEW HISTORY

BMJ Open publishes all reviews undertaken for accepted manuscripts. Reviewers are asked to complete a checklist review form (**http://bmjopen.bmj.com/site/about/resources/checklist.pdf**) and are provided with free text boxes to elaborate on their assessment. These free text comments are reproduced below.

ARTICLE DETAILS

**TITLE (PROVISIONAL)**	Digital auscultation as a novel childhood pneumonia diagnostic tool for community clinics in Sylhet, Bangladesh: protocol for a cross-sectional study
**AUTHORS**	Ahmed, Salahuddin; Mitra, Dipak; Nair, Harish; Cunningham, Steven; Khan, Ahad; Islam, ASMD Ashraful; McLane, Ian; Chowdhury, Nabidul Haque; Begum, Nazma; Shahidullah, Mohammod; Islam, Muhammad Shariful; Norrie, John; Campbell, Harry; Sheikh, Aziz; Baqui, Abdullah; McCollum, Eric D.

This article was not externally reviewed at BMJ Open. Protocols that have been independently assessed prior to submission to BMJ Open are usually fast-tracked to publication on the grounds that further substantial changes will not be possible. This independent assessment will usually be external, independent review for both a substantial grant award from a non-commercial or government funder and ethics approval.
